# Piglet gut microbial shifts early in life: causes and effects

**DOI:** 10.1186/s40104-018-0308-3

**Published:** 2019-01-14

**Authors:** Robin B. Guevarra, Jun Hyung Lee, Sun Hee Lee, Min-Jae Seok, Doo Wan Kim, Bit Na Kang, Timothy J. Johnson, Richard E. Isaacson, Hyeun Bum Kim

**Affiliations:** 1 Department of Animal Resources Science, Dankook University, Cheonan, 31116 South Korea; 2 National Institute of Animal Science, Rural Development Administration, Cheonan, 55365 South Korea; 3 Abbvie Bioresearch Center, Abbvie, Worcester, MA 01605 USA; 4 Department of Veterinary and Biomedical Sciences, University of Minnesota, St. Paul, MN 55108 USA

**Keywords:** Microbial diversity, Next generation sequencing, 16S rRNA, Swine microbiota, Weaning

## Abstract

The gut microbiome has long been known to play fundamentally important roles in the animal health and the well-being of its host. As such, the establishment and maintenance of a beneficial gut microbiota early in life is crucial in pigs, since early gut colonizers are pivotal in the establishment of permanent microbial community structures affecting the health and growth performance of pigs later in life. Emphasizing this importance of early gut colonizers, it is critical to understand the factors impacting the establishment of the piglet gut microbiome at weaning. Factors include, among others, diet, in-feed antibiotics, probiotics and prebiotic administration. The impact of these factors on establishment of the gut microbiome of piglets at weaning includes effects on piglet gut microbial diversity, structure, and succession. In this review, we thoroughly reviewed the most recent findings on the piglet gut microbiome shifts as influenced by weaning, and how these microbiome changes brought about by various factors that have been shown to affect the development of microbiota in piglets. This review will provide a general overview of recent studies that can help to facilitate the design of new strategies to modulate the gut microbiome in order to enhance gastrointestinal health, growth performance and well-being of piglets.

## Background

With the rapid transformational changes in molecular technologies and the development of “omics” strategies, the pig gastrointestinal (gut) microbiota has been intensively studied [1] and it is widely known to perform fundamentally important roles in health and well-being of animals [2, 3]. The gut microbiota provides the pig with many functions including improved energy harvesting capacity, the production of volatile fatty acids, production of vitamin K, cellulose fermentation, and enhanced resistance against pathogenic bacteria [1, 3, 4]. As this field of study continues to expand, it is clear that new roles and relationships between the gut microbiome and growth performance of the animal are yet to be discovered.

The pig gastrointestinal tract (GIT) harbors a diverse and complex microbial community. The total number of bacteria in the pig colon has been estimated to be 1 × 10^10^ - 1 × 10^11^ per gram of gut content [5]. Interestingly, the gut of neonates prior to birth is believed to be devoid of microbes but rapidly undergoes a remarkable shift from an essentially germ-free state to an extremely dense microbial population that eventually experiences a microbial succession and establishes an adult-like microbial community [2, 6]. The gut microbial composition and ecological succession of the intestinal microbiota in early life is shaped by a number of complex internal and external factors. For instance, dietary change, probiotics and prebiotics administration, and supplementation of in-feed antibiotics play important roles in shaping the gut microbial community of piglets [7–9]. As such, weaning at early stages of life is an important time of transition and stress in animals. Therefore, understanding of the dynamics of the pig gut microbiota during the weaning transition is of interest as it influences the overall health and growth performance of pigs.

In this review, we will discuss the most relevant findings of recent microbiome studies focusing on the analysis of the early-life pig gut microbiome as influenced by various factors, including diet, probiotics supplementation, and in-feed antibiotics, all of which have been shown to affect the development of microbiota in weanling pigs. Knowledge described in this review will facilitate in the development of new feeding strategies to assist in the establishment and maintenance of a beneficial gut microbiota of the piglets early in life. Here we provide a general overview of the recent studies about the intestinal microbiome of piglets at weaning.

### Weaning transition and influencers on the piglet gut microbial compositions

In pig production, weaning is an abrupt separation of pigs from the sow and it is characterized by stressful changes that can contribute to intestinal and immune dysfunctions leading to decreased piglet health and growth performance [10]. While weaning practices varies among swine producers, weaning age on commercial pig farms has been decreasing steadily with age less than 21 days. Generally, piglets are exposed to a number of stressors during the weaning transition, with among the most important being the abrupt change in diet from milk to plant-based solid feed. Weaning causes physiological changes in the structure and function of the intestine [11]. Moreover, the gut microbiota of young piglets undergoes a very quick ecological succession upon induction of various factors during the weaning period. Changes to the composition of resident commensal bacteria during the transitional periods in the piglet is referred to as a microbial shift [1]. This certainly occurs during weaning in piglets. One of the major factors influencing shifts in the microbiota in piglets during weaning is the abrupt change in diet from simple to more complex nutrient sources, which may affect absorption capacity of the small intestine, and likely influences growth and feed efficiency. The weaning period is critical as piglets are exposed to thousands of new bacterial species, which may play substantial roles in establishing an adult-like microbiota later in life [12]. Early-life microbial exposure is of particular importance to growth, development of immune system and health [13]. In addition, the establishment of a beneficial microbiota is important during the weaning stage because piglets still have an immature immune system and they are dependent on sow’s milk to prevent colonization and overgrowth of opportunistic pathogens [14]. Therefore, it is necessary to better understand the gut microbial succession during the weaning transition, and how diverse factors influence piglet gut microbial shifts in association with the enhance gastrointestinal health, growth performance and well-being of piglets [15].

In the recent years, several studies have explored the possible links between the intestinal microbiota composition of piglets and its association with growth performance and health, with research focus on the major factors thought to shape the piglet gut microbiota (diet, antibiotics, probiotics, prebiotics, and synbiotics) [3, 16]. Below, we will discuss the relative importance of the factors affecting the intestinal microbiota in piglets early in life and its implication for swine nutritional strategies to improve health and performance of pigs.

### The pig gut microbiota in early life and its influence on post-weaning infections

The weaning period is a crucial stage in the life of pigs, as the gut microbial composition and the immune system are still developing, making the pigs susceptible to pathogens leading to post-weaning diarrhea [15]. Diarrhea has been the leading and increasing cause of mortality in the swine industry. Interestingly, there is an increasing evidence in recent years suggesting the gut microbiota contributes in the development of diarrhea in weaned piglets, not just specific pathogens [13]. It has been recognized that gut microbiota provides protection against pathogens by regulating the immune response of the host. For example, segmented filamentous bacteria (SFB) or *Candidatus * Arthromitus, which are known modulators of the mammalian immune system, have been identified as a major group of bacteria in the terminal ileum of weaning piglets [17, 18]. However, there is limited information on the early-life microbial community structure and function of the gut microbiota and its role in pathogenesis of post-weaning diarrhea in pigs. A recent metagenomic analysis of the fecal microbiota in diarrheic piglets has revealed that diarrhea was associated with increase in the relative abundance of *Prevotella*, *Sutterella*, *Campylobacter* and Fusobacteriaceae [19]. Another report assessed the potential early gut microbiota composition of piglets as an indicator of susceptibility to post-weaning diarrhea [13]. In that study, piglets were weaned in poor housing conditions to challenge their susceptibility to diarrhea. When compared to diarrheic piglets, the gut microbiota of healthy piglets had higher abundance of Prevotellaceae*,* Lachnospiraceae*,* Ruminococcaceae and Lactobacillaceae [13]. Results from these studies suggest that the gut microbial composition could be used as a biomarker to predict health status of piglets. However, further work is necessary to understand the mechanisms of action of the gut microbial community in protection, and the development of clinical interventions for better gut health of the piglets.

Importantly, during the weaning transition piglets experience an immediate but transient drop in feed intake or anorexia, which contributes to intestinal inflammation [20, 21]. In a recent study, the mechanisms by which the gut inflammation contributes to imbalance of the microbiota has been proposed [22]. Under intestinal inflammatory conditions, host response results in production of reactive oxygen species such as nitric oxide (NO) that is rapidly converted to nitrate (NO_3_^−^) when released in the intestinal lumen [15, 22, 23]. The nitrate-rich environment is conducive for the growth of Enterobacteriaceae that encodes for nitrate reductase genes [23, 24]. Of note, some pathogens within the family Enterobacteriaceae, namely *Salmonella enterica* serovar Typhimurium and enterotoxigenic *E. coli* (ETEC), induce intestinal inflammation in pigs which disrupts the microbiome composition [25, 26]. For example, in a piglet model of *Salmonella* Typhimurium infection, Arguello et al. [27] observed that there was a decrease in the population of desirable bacteria such as *Bifidobacterium* and *Lactobacillus* with an increase in pathogenic bacteria, *Citrobacter* and a depletion in anaerobic bacteria namely *Clostridium*, *Ruminococcus* or *Diallister* at the ileum mucosa of weaned piglets. Therefore, intestinal inflammation associated with weaning initiates disturbances in the gut microbiota, which favors the growth of enteropathogenic bacteria especially Enterobacteriaceae. However, further research is warranted to understand the impact of intestinal inflammation on gut microbiota disruptions in the post-weaning piglets.

### Host genetic effects on piglet gut microbiota

Host genetics has been considered a major factor shaping the intestinal microbiota of mice and humans [28, 29]. Previously, the work of Ochman et al. [30] revealed that host genetics was the dominant factor that influenced the distal gut microbial communities within the primate gut over evolutionary timescales. The gut microbial composition of pigs is likely also shaped by host genetics. Several studies have reported the impact of host genetics on the development of gut microbiota in piglets at the early stages of life. Recently, a study by Pajarillo et al. [9] demonstrated an interaction between the fecal microbial community and pig breed using pyrosequencing of the 16S rRNA gene. This study was conducted to investigate the differences and similarities in fecal microbial communities among the three purebred pig lines (Duroc, Landrace and Yorkshire), and to discover possible links between microbiota and host genetics. At the phylum level, majority of the sequences were classified as the phyla Firmicutes and Bacteroidetes regardless of the pig breed which is congruent with previous studies. However, the proportion of bacteria belonging to phylum Firmicutes was more abundant in Landrace pigs than in Duroc and Yorkshire pigs [9]. At the genus level, *Prevotella*, *Blautia*, *Oscillibacter*, and *Clostridium* were identified in all fecal samples regardless of the pig breed. In addition, *Catenibacterium*, *Phascolarctobacterium*, and *Subdoligranulum* were more abundant in Duroc pigs, while *Dialister* was more abundant in Yorkshire pigs. It was suggested that the microbiome of Yorkshire and Landrace pigs were similar but distinct to that of Duroc pigs, and that this may be due to gene pool similarities between Yorkshire and Landrace pigs. Therefore, the differences in the composition of the gut microbiota of pigs may be attributed to host genetics since all pigs were raised under the same controlled environment and fed with similar diets. However, the authors suggested that other environmental factors, such as pen and seasonal effects must also be considered to link differences in gut microbiota with host genetics [9].

Another study by Bian et al. [7] used a piglet-cross fostering model as a tool to study the effects of host genetics and other factors such as diet, maternal effects, and the environment on the development of pig gut microbiota from birth to 7 weeks of life. They used newborn piglets of two different breeds of pigs including Meishan and Yorkshire pigs. In that study, breed strongly affected the composition of several bacterial taxa during the suckling period. For example, the Meishan piglets had a higher population of family Fusobacteriaceae and a lower relative abundance of Erysipelotrichaceae as compared with the Yorkshire. At the genus level, the population of *Bacteroides* was higher in Yorkshire than in the Meishan piglets. However, the bacterial composition was not significantly caused by the pig breed but by dietary change after weaning [7].

The pig genetic background can be considered as a predisposing factor to gut infection resulting in gut microbial shifts. The ETEC expressing the F4 fimbriae (ETEC F4) has been known to be one of the major causes of diarrhea in neonatal and post-weaned piglets [31, 32]. In pigs, the susceptibility to ETEC F4 diarrhea is determined by the intestinal F4 receptor, that allows the bacterium to adhere to the intestinal tract [32, 33]. Several candidate genes of ETEC F4 receptor including the Mucin 4 (*MUC4*) gene were investigated [34, 35]. Furthermore, in pigs, alpha-(1,2) fucosyltransferase (*FUT1*) gene has been recognized for its properties in controlling the intestinal expression of ETEC F18 receptors [36]. It has been proposed that piglets of the *FUT1* genotype can be classified according to susceptibility to F18 fimbriated *E. coli* with genotype *FUT1*^*AA*^ piglets as resistant and piglets with the *FUT1*^*AG*^ and *FUT1*^*GG*^ genotypes as susceptible [37]. In a recent study, Riis et al. [38] showed that piglets of *FUT1*^*AG*^ genotype had higher number of hemolytic bacteria and Enterobacteriaceae compared to *FUT1*^*AA*^ piglets. Thus, host genetics play an important role in susceptibility or resistance of pigs to post-weaning infections resulting in microbial shifts. However, systematic studies are still required to exactly understand the principles dictating how host genetic factors shape the complex microbial communities in pig.

These results demonstrate that host genetics can be characterized as a major internal factor that shapes the intestinal microbiota of piglets, which is evident in early life of pigs. Nevertheless, further research is needed to fully decipher the impact of host genetics on the early colonization and development of gut microbiota of piglets and its subsequent impact on growth, health and performance.

### Effects of diet on the piglet gut microbiota

**Fig. 1:**
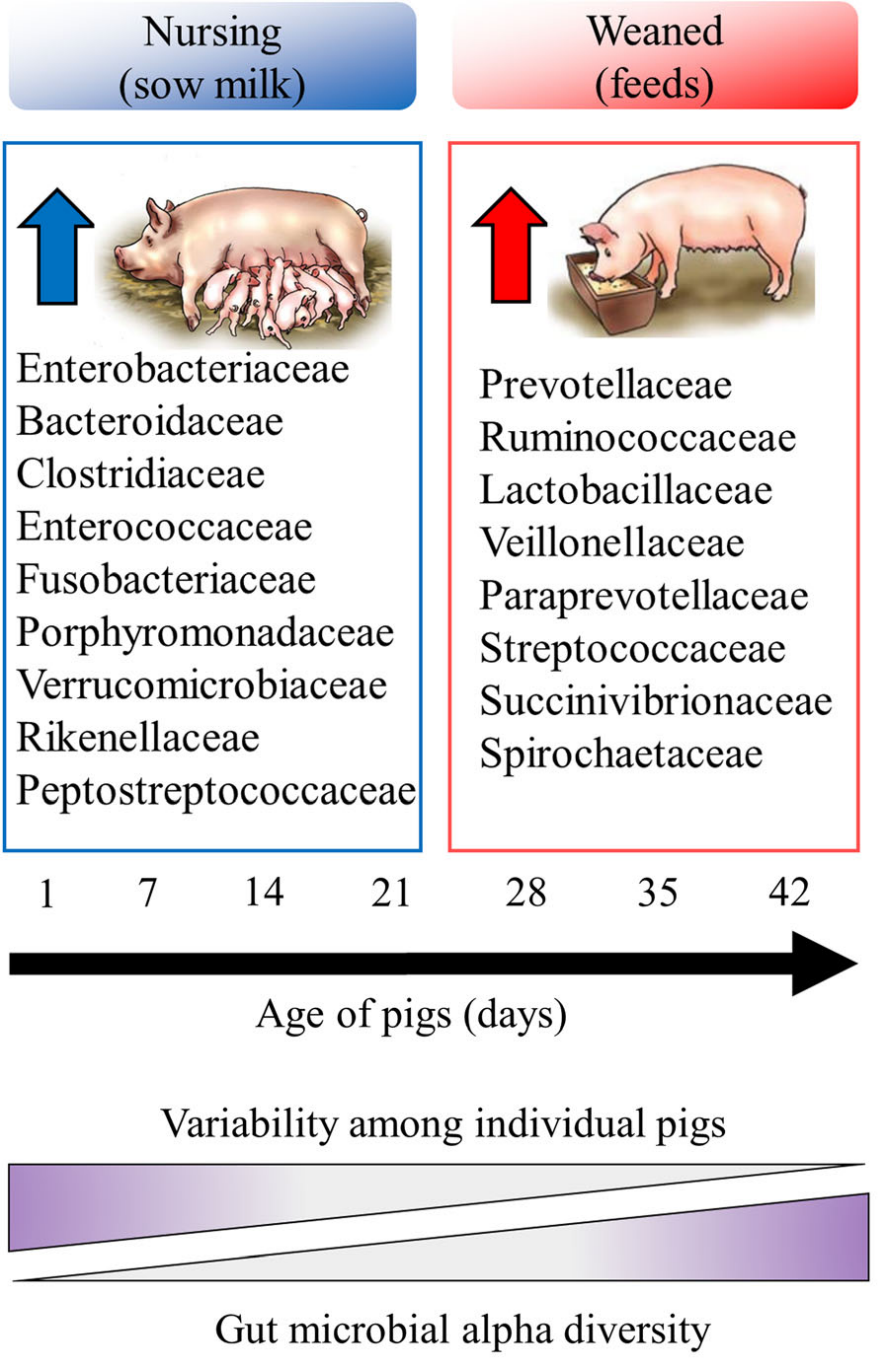
Bacterial alpha diversity and composition shifts as influenced by diet. Alpha diversity indices of the piglet gut microbiota increase while the variability of the microbiota among individual piglets decreases as the piglets age. Significant differences between mean proportions of bacterial taxa at the family level in nursing and weaned pig fecal samples were indicated as the blue and red up arrows, whose relative abundances were higher in nursing and weaned piglets, respectively. Figure was modified with permission from the article by Frese et al. [39]

The first year of life is important in establishing the gut microbiota of animals and it is strongly affected by diet [39]. Several studies reported effects of dietary change on the piglet gut microbiome, overall health, and growth performance. Frese et al. [39] used 16S rRNA gene and whole metagenome sequencing (WMS) to characterize the fecal microbiomes of piglets from birth to 7 weeks of age. In this study, they evaluated the effects of dietary change from sow’s milk to a plant-based starter diet on pig gut microbial structure, function, and ecological succession. Figure 1 shows the schematic diagram of piglet gut microbial alpha diversity and the changes in microbial taxa at the family level from birth through weaning, as influenced by diet. Alpha diversity of microbiota is a measure of within-sample diversity. It can be concluded that there was a precise difference in alpha diversity of piglet gut microbiota before and after weaning in piglets. Alpha diversity of the piglet gut microbiota increased concurrently with the dietary change from sow’s milk to plant based starter diet. At the family level, relative abundances of Bacteroidaceae and Enterobacteriaceae declined over time while those of Lactobacillaceae, Ruminococcaceae, Veillonellaceae, and Prevotellaceae increased in weaned piglets. These observations are in agreement with previous dietary studies investigating differences in animal and plant-based diets [6, 12].

In a recent study, Guevarra et al. [40] reported the significant increase in the genus *Prevotella* and *Lactobacillus* after weaning compared to those of suckling piglets. It has been known that members of genus *Prevotella* metabolize plant-derived non-starch polysaccharides to short-chain fatty acids (SCFAs) [41]. *Prevotella* spp. have also been known to degrade polysaccharides in plant cell wall by producing enzymes, such as β-glucanase, mannase, and xylanase [42]. In addition, Lactobacilli have been known to metabolize carbohydrates including oligosaccharides and starch, which are fermented in the large intestine to SCFAs by lactobacilli, then be utilized by the pigs [43]. Even though further studies are required to elucidate the influence of diet and host-microbial crosstalk in piglet health and performance, these findings suggest that the pig gut microbial community structure and functional capacities were oriented to deal with stresses caused by dietary change during the weaning transition.

Other studies also have contributed to our knowledge on how the gut microbiome responds to changes in diet during nursing and the weaning transition in piglets. A recent study by Zhang et al. [44] indicated that a moderate increase in dietary fiber influenced the microbial composition in the gut of suckling piglets. They aimed to evaluate the effects of dietary fiber sources on the gut microbiota in the large intestine of the piglets during the suckling period. In that study, suckling piglets were fed a diet rich in fiber, particularly alfalfa which is rich in insoluble fiber (cellulose) but also has soluble fibers including fructans and pectins [45]. They hypothesized that a moderate addition of dietary fiber during the suckling period may affect the gut microbiota. Interestingly, results from their study showed that a diet rich in alfalfa decreased the abundance of *Streptococcus suis*, a known pathogen that induces bacterial mortality of piglets after weaning [46]. In addition, the diet containing alfalfa increased the abundance of *Coprococcus eutactus* in the distal colon at weaning. *Coprococcus eutactus* belongs to the *Clostridium* group with butyrate-producing ability. Alfalfa meal has also been shown to alternate gut microbiota in piglets resulting in beneficial effects on the gut health [47]. Although, the effects of adding fiber to diets on growth performance in piglets were inconsistent, these studies suggest that dietary supplementation with alfalfa has the potential to improve gut health in piglets and to increase the abundance of beneficial microbes.

Overall, these studies and many other studies [48–52] suggest that gut microbiota can be modulated by various dietary components such as dietary fiber, dietary protein, and minerals. However, further research is needed to fully understand the mechanisms involved in the interactions between diet composition and the piglet gut microbiota.

### Effects of probiotics, prebiotics and synbiotics on weaned piglet gut microbiota

The Food and Agricultural Organization of the United Nations (FAO) and the World Health Organization (WHO) define probiotics as live microorganisms that, when administered in adequate amounts, confer health benefits on the host [53]. Probiotics are one of the functional foods that link diet and health. In 2007, Roberfroid defined a prebiotic as “a selectively fermented ingredient that allows specific changes, both in the composition or activity in the gastrointestinal microflora that confer benefits upon host well-being and health” [54]. Over the past few decades, probiotics and prebiotics or their combination (also known as synbiotics) have been the subject of many research studies because of their potential therapeutic and preventive health benefits to animals [55]. Previous reports have shown that probiotics and prebiotics have a broad range of beneficial effects in pigs including fortification of the intestinal barrier function [56, 57], reduction of diarrhea duration and severity [28–30], inhibition of pathogenic bacteria [58], and immunological development [57, 59].

**Fig. 2:**
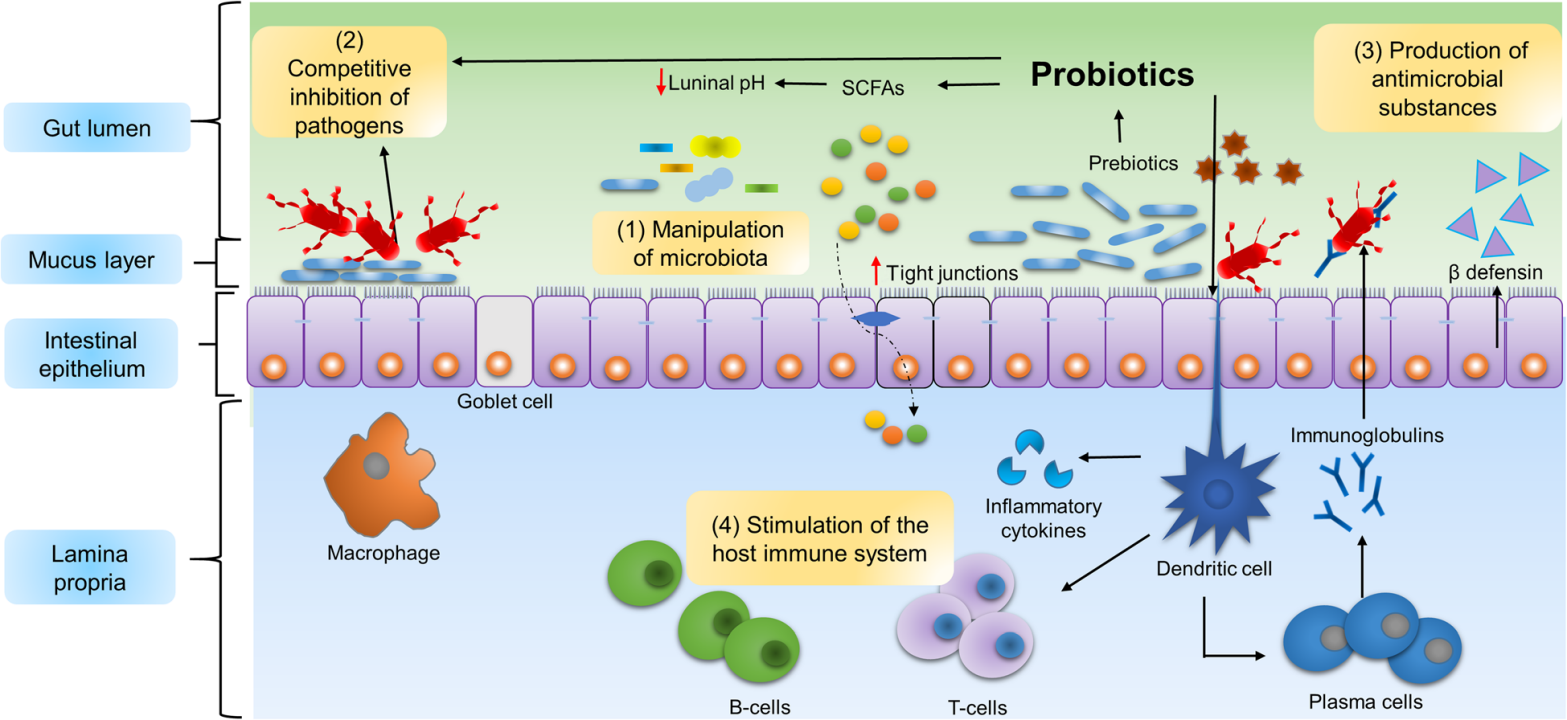
The potential mechanisms by which probiotics affect intestinal microbial ecology. Probiotics may act through the following mechanisms: (1) manipulation of the microbiota by changing luminal pH, (2) competitive inhibition of pathogen, (3) production of antimicrobial substances and (4) stimulation of the pig’s immune system

The mechanisms associated with the beneficial effects of prebiotics and probiotics includes manipulation of intestinal microbial communities. Because prebiotics are readily available fermentation sources for probiotics, prebiotics may improve the survival of concurrently administered probiotic strains resulting in advantages to the host that the live microorganisms offer. Consequently, prebiotics help probiotics to manipulate intestinal microbial communities by lowering luminal pH and stimulating the host immune system leading to the production of antimicrobial substances. In addition, probiotics influence gut microbial ecology by inhibiting pathogens (Fig. 2).

A recent study by Chae et al. [8] showed using 454 pyrosequencing of the 16S rRNA gene that there were shifts in the composition of the fecal microflora in response to the administration of probiotics, prebiotics and synbiotics in weaned piglets. In that study, seventy-nine healthy piglets were used to investigate the effects of the prebiotic lactulose, the probiotic *Enterococcus faecium* NCIMB 11181, or their synbiotic combination. At the phylum level, the majority of microbes (> 90%) belonged to the phyla Firmicutes and Bacteroidetes, regardless of the feed additive. However, prebiotic lactulose supplementation significantly increased the Firmicutes-to-Bacteroidetes ratio. In weaned piglets, the gut microbiota of heavier piglets had a higher Firmicutes-to-Bacteroidetes ratio than that of piglets with lighter weight [60]. Thus, it has been speculated that lactulose promoted the growth of some bacterial groups in the phylum Firmicutes, which may promote various metabolic activities when complex plant-based diets were fed. At the family level, Lactobacillaceae numbers were increased after administration of the prebiotic lactulose, the probiotic *Enterococcus faecium* NCIMB 11181, or their synbiotic combination. This suggests that there is a complementary effect of probiotics and prebiotic administration triggering the growth of Lactobacillaceae. Moreover, the proportions of Enterobacteriaceae were markedly decreased in all treatment groups. This is favorable for animal production since piglets have high proportions of this family are more prone to diseases [8, 15]. At the genus level, the proportions of *Oscillibacter*, *Clostridium* and *Lactobacillus* were highest in probiotics, probiotics and synbiotic-treated groups, respectively. It was speculated that the increase in *Lactobacillus* population in the synbiotic group might be depending on the used strain of *Lactobacillus* because an administration of *L. plantarum* and lactulose in a different study did not result in significant proliferation of *Lactobacillus* [61]. In addition, it was observed that the probiotic group had the lowest relative abundance of *Escherichia*. These findings suggest that the influence of synbiotics on gut microbiota of piglets may differ functionally and mechanistically compared to individual administration of probiotics or prebiotics [8].

The effects of probiotic *Propionibacterium freudenreichii* (PF) supplementation on the microbial community of the colon was analyzed using 16S rRNA pyrosequencing of the samples from 16 piglets [62]. In this study, there was a significant decrease in the population of potential pathogenic bacteria belonging to the family Porphyromonadaceae in the PF treated group. It was speculated that the decrease in Porphyromonadaceae in the PF-treated group might depend on the production of bacteriocins and short-chain fatty acids (SCFAs) by the PF. These findings suggest that probiotic supplementation in feed has the ability to decrease potentially pathogenic bacteria in the gut of piglets after weaning. Overall, results from this study suggest that the piglets are susceptible to pathogenic bacteria invasion during weaning, however, inclusion of probiotics in a diet induced positive response to growth performance by promoting overall enteric health of weaned piglets.

Many studies suggested that probiotics, prebiotics and their synbiotic combination exerted beneficial effects on piglets through the modulation of gut microbiota. However, there is a need to clarify the underlying mechanisms of probiotic supplementation on the intestinal microbial composition because other studies reported no effect of probiotic supplementation on the gut microbial communities [63].

### Effects of antibiotics on piglet gut microbiota before and after weaning

For the past six decades, antibiotic growth promoters (AGPs) have been used to promote piglet growth starting at weaning. AGPs, unlike therapeutic applications of antimicrobials, are provided to pigs continuously at much lower dosages resulting in enhanced pig production. When AGPs are first administered to piglets early in life, their microbiomes are composed of bacteria that are more vulnerable to changes. Thus it was speculated that administration of AGPs could enhance growth performance of piglets by modulating the gut microbiome [64]. AGPs such as tylosin, bacitracin, virginiamycin, and chlortetracycline have been used in piglets to promote growth performance through increased feed conversion and weight gain that leads to healthier animals [64]. However, there are increasing concerns related to the development of antibiotic-resistant bacterial strains and antibiotic residues in meat products and animal feces [65, 66]. Moreover, the potential adverse impact of these antibiotic resistance genes to human health has been increasing. Consequently, the use of antibiotics as growth promoters in swine has been banned in many developed countries including those of the European Union (EU), the USA, and Canada. While the mechanisms by which antibiotics enhance the growth of livestock animals are unclear, many studies have proposed that growth promotion of livestock animals was due in part to the alteration of their gut microbiota [67]. Several studies have been conducted to evaluate the effect of antibiotics on the pig gut microbiota [64, 68]. In addition, piglets are frequently exposed to antibiotics early in life primarily to prevent outbreaks of intestinal and respiratory diseases, thus it is important to understand how antibiotics affect the intestinal microbiota.

In a recent study, Kim et al. [64] reported the effects of antibiotic growth promoter tylosin on swine microbiota using 454 pyrosequencing of V3 region of the 16S rRNA gene. They showed that intestinal microbiota of the piglets treated with tylosin had microbial population shifts as denoted by an increase in microbial succession rate and maturation of the gut microbiota. Moreover, tylosin caused microbial population shifts in both the abundant and less abundant species, as shown by quantitative analyses. At the genus level, that study showed that populations of *Lactobacillus*, *Sporacetigenium*, *Acetanaerobacterium*, and *Eggerthella* were more abundant in tylosin treated pigs as compared to the untreated ones. It has been suggested that tylosin may hasten the maturation and development of microbiota in pigs to adult-like gut microbial community [64].

Schokker et al. [68] investigated the effects of early-life exposure to antibiotics on the diversity of the gut microbiota and immune system development in 4-day old pigs. Using microarray-based analysis, a profile of the piglet gut microbiota in response to tulathromycin treatment was obtained. At the phylum level, the intestinal microbiota for all piglets was primarily composed of the phyla Firmicutes, Proteobacteria, Bacteriodetes, Spirochaetes, and Actinobacteria irrespective of tulathromycin treatment. Interestingly, a single dose of antibiotic administered at the pre-weaning period was able to modulate the gut microbiota for a prolonged period. This indicates that the composition of the microbiota may not immediately revert to its primary state following antibiotic treatment, and that long-lasting effects on gut microbiome structure can ensue following such treatment. In that same study, antibiotic treatment increased the relative abundance of anaerobic bacteria including *Bifidobacterium*, *Eubacterium*, *Faecalibacterium prausnitzii*, and *Solobacterium moorei*. However, tulathromycin decreased the relative abundance of facultative bacteria such as *Staphylococcus aureus*.

These studies demonstrate how antibiotics may shape the intestinal microbiota of pigs in early life, and strongly suggest a link between antibiotic supplementation and gut microbiota dysbiosis in early life of pigs. In summary, recent published research data clearly showed that subtherapeutic use of antibiotics in pig production improves growth rate, reduces morbidity and mortality and improves the overall health in pigs. However, subtherapeutic use of antibiotic growth promoters raises concerns such as microbial dysbiosis that can have long-lasting effects. Moreover, antibiotics used in the swine production can contribute in the dissemination of antibiotic resistance genes in the environment and antibiotic residues in the human food chain which may bring potential impacts on human health. Nevertheless, the mechanism by which antibiotic-induced microbial dysbiosis influences growth of the animal needs further research for the development of antibiotic alternatives that mimic these changes.

### Functional metagenomics approach for better understanding of the young piglet gut microbiome

The advent of modern molecular techniques has profoundly expanded our knowledge of the swine gut microbiota. In the recent years, amplification of the 16S rRNA gene coupled with next-generation sequencing technologies have enabled us to intensively explore the gut microbial composition of pigs [1]. While 16S rRNA gene sequencing uncovers the complex taxonomic profile of a microbial community, it is also important to understand the functional capacities of the early-life piglet gut microbiota and its potential contributions to the physiology and metabolism in the pig’s GIT. This can now be accomplished predictively through whole-metagenome sequencing. [40].

**Fig. 3:**
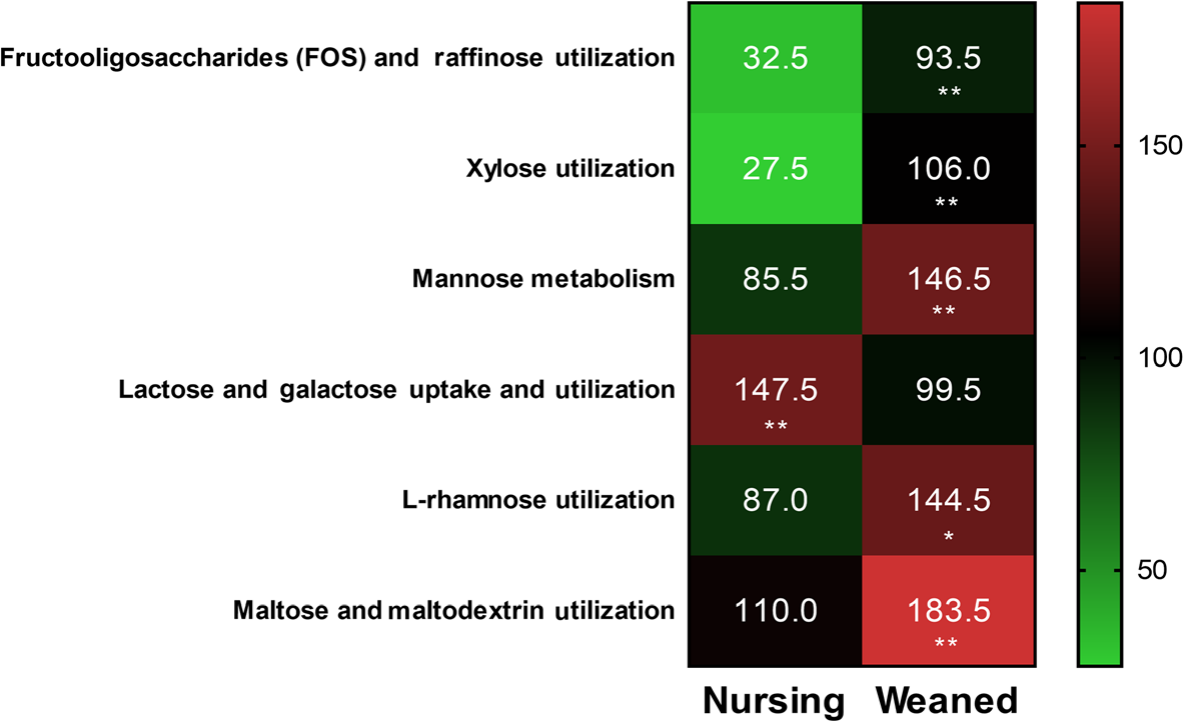
The functional capacities of the microbiome between nursing and weaned piglets in association with carbohydrate metabolism as revealed by whole metagenome shotgun sequencing. The scale bar indicates normalized abundance of the level 3 SEED subsystem classified reads associated with carbohydrate metabolism. The [*P* < 0.001], [*P* < 0.01] and [*P* < 0.05] were indicated as [***], [**] and [*], respectively

The taxonomic compositions and functional capacity of the piglet gut microbiome during the weaning transition has been reported previously [69]. Frese et al. 2015 [39] examined the bacterial metagenome of piglets from birth through weaning using WMS approach. In particular, metagenomic sequencing revealed that nursing pig metagenome was enriched in proteins involved in utilization of milk-derived glycans such as sialidase (EC 3.2.1.18) and beta-hexosaminidase (EC 3.2.1.52) which are contributed primarily by *Bacteroides*. By comparison, the weaned piglet gut microbiome was enriched in proteins involved in degradation of plant-derived substrates including β-xylosidases (EC 3.2.1.37), endo-1,4-β-xylanases (EC 3.2.1.8), and α-N-arabinofuranosidases (EC 3.2.1.55) which are contributed by various members of the gut microbiota [39]. A more recent study by Guevarra et al. [40] provided new insight into the potential contribution of early-life gut microbiome in association with pig health and nutrition using WMS approach. In their study, a SEED-based functional analysis of the piglet gut microbiome revealed that gene families mapping to carbohydrate metabolism were significantly higher in the weaned piglets (*P* < 0.05). When the pigs were weaned and complex plant-based feeds were introduced, the carbohydrate composition of the porcine diet abruptly increases. When such complex plant-derived glycans enter the gut, the functional capacities of the microbiota shift. The abundance of genes mapping to carbohydrate metabolism categories including “xylose utilization”, “mannose metabolism” and “L-rhamnose utilization” associated with components of plant-derived polysaccharides was significantly more prevalent in weaned pigs. In contrast, the microbiome of the nursing piglet had significantly enriched gene families associated with “lactose and galactose uptake and utilization” correlating with lactose being the principal sugar in porcine milk (Fig. 3).

In another report, a WMS approach was used to investigate the functional diversity of the fecal microbiota in diarrheic piglets [19]. In this study, the gut microbiome of piglets with diarrhea was enriched in functional gene abundance associated with the bacterial ribosome, which was mainly contributed by *Prevotella.* Furthermore, their study revealed that piglet diarrhea is associated with an enrichment in genes involved in bacterial polyamine transport, amino acid transport, and two-component regulatory system. These functional genes identified in diarrheic piglets indicate that *Prevotella* may play important roles to combat post-weaning diarrhea and *Prevotella* may contribute in nutrient transport and uptake in the piglet gut ecosystem [19].

The advancement of metagenomic methods have leveraged our current understanding of the dynamics of the pig gut microbiome through WMS in association with abrupt dietary change and the potential contribution of the microbiome in shaping the piglet health and growth performance. Characterization of both microbial structure and functional capacities of the piglet gut microbiome in early-life could reveal potential biomarkers and therapeutic targets for prevention of post-weaning infections and eventually improvement in health and growth performance.

## Conclusions

The intestinal microbiota is known to play major roles in the development of immune system and the overall health of piglets. The composition and diversity of the piglet gut microbiota during the early stages of life are not fixed, and are affected by various factors including diet and use of antibiotics and probiotics. Weaning and/or the change to consumption of solid feed marks an important and remarkably mosaic period of microbial shifts within the pig gut. Irrespective of weaning, piglet gut bacterial diversity increases, while variability caused by idiosyncratic effects is reduced, as piglets age. Findings from these studies should facilitate the development of evidence-based strategies to assist in the establishment of healthy gut microbiota of piglets during early stages of life, and to maintain beneficial microbes that are important to enhance the gastrointestinal health of piglets. Nevertheless, there is still an overall paucity of knowledge about the piglet gut microbiota during the weaning transition. There is an eminent need to explore which bacterial species are really beneficial to piglets because we do not fully understand the functional roles of each species in pigs. It is worth noting that the work performed thus far is mainly descriptive, and studies are greatly needed that employ metagenomics, metabolomics, and metatranscriptomics based approaches, at the same time testing specific hypotheses related to mechanism and function. Such studies will help to further elucidate the role of the piglet gut microbiome in overall health and development.

## Abbreviations

16S rRNA: 16S ribosomal ribonucleic acid
AGP: Antibiotic growth promoter
ETEC: Enterotoxigenic *Escherichia coli*
FAO: Food and Agricultural Organization
FUT1: Alpha-(1,2) fucosyltransferase
GIT: Gastrointestinal tract
MUC4: Mucin 4
NCIMB: National Collections of Industrial, Marine and Food Bacteria
NO: Nitric oxide
SCFA: Short-chain fatty acid
SFB: Segmented filamentous bacteria
WHO: World Health Organization
WMS: Whole metagenome sequencing
